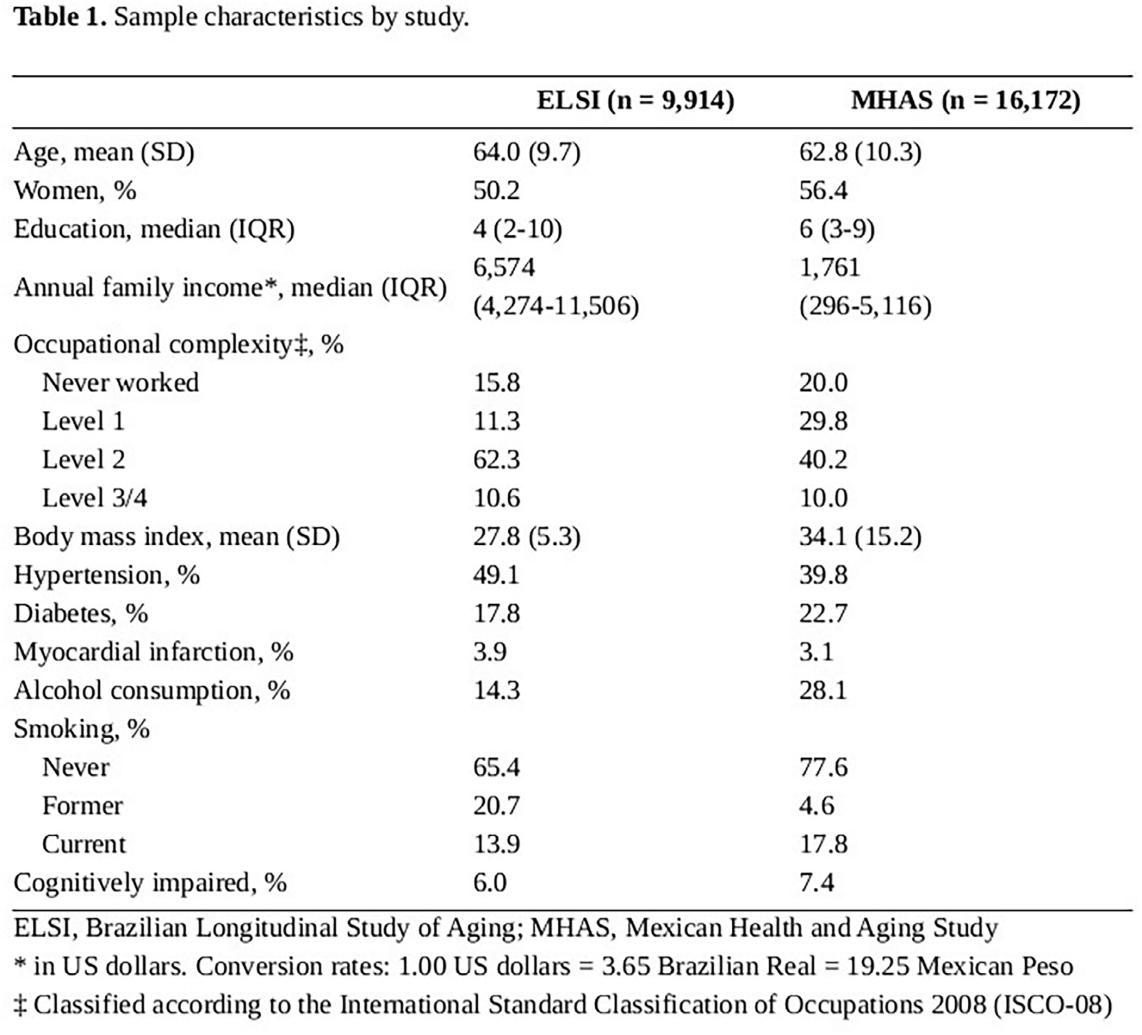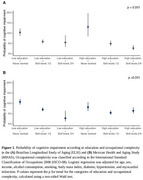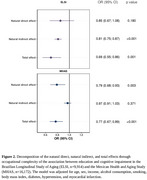# Associations of education and occupation combinations with cognitive impairment in Brazil and Mexico

**DOI:** 10.1002/alz70860_100388

**Published:** 2025-12-23

**Authors:** Natalia Gomes Goncalves, Lidia Lack, Gabriela Mininel de Medeiros, Jaqueline Contrera Avila, Laiss Bertola, Maria Fernanda Lima‐Costa, Cleusa P Ferri, Rebeca Wong, Claudia Kimie Suemoto

**Affiliations:** ^1^ University of São Paulo Medical School, São Paulo, SP, Brazil; ^2^ University of Sao Paulo Medical School, Sao Paulo, SP, Brazil; ^3^ University of São Paulo, São Paulo, SP, Brazil; ^4^ University of Massachusetts Boston, Boston, MA, USA; ^5^ Universidade Federal de São Paulo (UNIFESP), São Paulo, São Paulo/SP, Brazil; ^6^ Fiocruz, Belo Horizonte, MG, Brazil; ^7^ University of Texas, San Antonio, TX, USA; ^8^ University of São Paulo Medical School, São Paulo, São Paulo, Brazil

## Abstract

**Background:**

Education and occupational complexity have been individually associated with better cognitive performance and lower odds of cognitive impairment, mostly in high‐income countries. Thus, it is important to investigate this association in the context of low‐ and middle‐income countries where there is greater variability in educational attainment and occupational complexity. We use nationally representative data from Brazil and Mexico to assess the associations of education and occupational complexity combinations with cognitive performance and to investigate if occupational complexity mediated the association between education and cognitive performance.

**Method:**

The sample included adults 50 years or older from the 2018‐2019 waves of the Brazilian Longitudinal Study of Aging (ELSI, *n* = 9,914) and the Mexican Health and Aging Study (MHAS, *n* = 16,172). Participants were classified as cognitively impaired or not impaired using a regression‐based approach compared to a normative sample. Educational attainment was categorized into low (≤8 years of education) and high (>8 years of education). Occupational complexity was defined using the International Standard of Classification of Occupations 2008 and categorized into never worked, skill levels 1/2, and skill levels 3/4. Linear regression models investigated the combined association of education and occupation with cognitive function. The mediation analysis used the counterfactual framework.

**Result:**

Participants of the ELSI had a mean age of 64.0±9.6 years, median (interquartile range) years of education of 4 (2‐10) years, 50.0% were women, 15.8% had never worked, and 6% were cognitively impaired (Table 1). Participants of the MHAS had a mean age of 62.8±10.3 years, median years of education of 6 (3‐9) years, 56.0% were women, 20.0% had never worked, and 7% were cognitively impaired (Table 1). Within the levels of education, there was a dose‐response with increasing occupational complexity in the association with cognitive function (*p* for trend: ELSI < 0.001; MHAS < 0.001) (Figure 1). Occupational complexity explained 57.8% of the relationship between education and cognitive function in the ELSI, but was not a mediator in the MHAS (Figure 2).

**Conclusion:**

Our results highlight the cumulative benefits of education and occupation on cognition, with occupational complexity's mediating role in the education‐cognition association differing between Brazil and Mexico.